# Bringing Geroscience to the Bedside: Leveraging Biomarkers of Aging in Clinical Research

**DOI:** 10.1093/geroni/igaf122.1669

**Published:** 2025-12-31

**Authors:** Sara LaHue

**Affiliations:** University of California, San Francisco, San Francisco, California, United States

## Abstract

Biomarkers of aging, including epigenetic and proteomic “clocks” and senescence-associated secretory phenotype (SASP) factors, offer powerful tools for potentially understanding and predicting outcomes in older adults, yet their application in patient-facing clinical research remains limited. In this talk, I will share practical lessons from integrating geroscience markers into patient cohorts, focusing on delirium and other complications that follow traumatic injuries in older adults, to show how aging biomarkers may both illuminate mechanisms of vulnerability and serve as predictors of adverse clinical outcomes. I will highlight strategies for measuring these markers in clinical settings and overcoming implementation challenges to demonstrate that geroscience biomarkers are not only feasible to integrate but also essential for advancing precision approaches to aging-related conditions.

